# COPD Risk Phenotypes in Older Smokers: Evaluation in GLI- and GOLD-Defined Respiratory Impairment

**DOI:** 10.1007/s00408-024-00757-4

**Published:** 2024-11-27

**Authors:** Abraham Bohadana, Pascal Wild, Ariel Rokach, Assaf Berg, Gabriel Izbicki

**Affiliations:** 1 Department of Medicine, Shaare Zedek Medical Center and Faculty of Medicine, Respiratory Research Unit, Pulmonary Institute, Hebrew University of Jerusalem, 12, Bayit Street, 91031 Jerusalem, Israel; 2 PW Statistical Consulting, 54000 Laxou, France; 3 Hadassah School of Medicine, Hebrew University of Jerusalem, Jerusalem, Israel

**Keywords:** COPD risk phenotype, GLI, GOLD, Elderly smokers, Misclassification, Spirometry

## Abstract

**Purpose:**

In aging populations, the Global Initiative for Obstructive Lung Disease (GOLD) spirometry threshold may misclassify normal spirometry as airflow limitation. The Global Lung Initiative (GLI) method provides age-adjusted criteria. We investigated how the use of GOLD or GLI thresholds in an algorithm affects the classification of elderly smokers into COPD risk phenotypes.

**Methods:**

Using a modified COPDGene algorithm, including exposure, symptoms, and abnormal spirometry, 200 smokers aged 60 years and older were classified into 4 mutually exclusive phenotypes: Phenotype A (no symptoms, normal spirometry; reference), Phenotype B (symptoms, normal spirometry; possible COPD), Phenotype C (no symptoms, abnormal spirometry; possible COPD), and Phenotype D (symptoms, abnormal spirometry; probable COPD). Abnormal spirometry was defined according to the GOLD or GLI criteria. A comparison was made between the GOLD- and GLI-defined phenotypes.

**Results:**

Using GLI criteria/cut-offs, 18.5% (*n* = 37) had phenotype A (no COPD), 42% (*n* = 84) had phenotype B (possible COPD), 7.5% (*n* = 15) had phenotype C (possible COPD), and 32% (*n* = 64) had phenotype D (probable COPD). Using GOLD criteria cut-offs, 14.5% (*n*-29) had phenotype A (no COPD); 31% (*n* = 62) had phenotype B, 11.5% (*n* = 23) had phenotype C (probable COPD), and 43% (*n* = 86) had phenotype D (probable COPD). Eight smokers with GOLD phenotype C were reclassified as GLI phenotype A, while 22 with GOLD phenotype D were reclassified as GLI phenotype B. Smokers identified as ‟probable COPD” by GOLD alone (potential false positives) had better spirometry results than those identified as ‟probable COPD” by both GOLD and GLI.

**Conclusion:**

The use of the GOLD threshold in an algorithm resulted in older smokers being classified into more severe COPD risk phenotypes compared to the GLI threshold. This suggests that GOLD may misclassify smokers with less affected phenotypes as having respiratory impairment, potentially leading to unnecessary and harmful treatments.

## Introduction

Chronic obstructive pulmonary disease (COPD) is a major global health problem [1, 2]. The Global Initiative for Chronic Obstructive Lung Disease (GOLD) guidelines recommend a ratio of forced expiratory volume in one second to forced vital capacity (FEV1/FVC) < 0.7 for diagnosis [3], but smokers without airflow obstruction may present with symptoms and structural changes similar to those seen in spirometrically diagnosed COPD [4, 5]. Some may progress to COPD via a predominant airway pathway that progresses through impaired spirometry with preserved ratio spirometry (PRISm) (i.e., low FEV1 with normal FEV1/FVC) to GOLD stages [6].

Recognizing these discrepancies, the large 10-year longitudinal genetic epidemiology study (COPDGene) [7] demonstrated that a comprehensive approach including smoking exposure, symptoms, assessment of structural abnormalities, and spirometry can better define COPD and predict progression to airflow obstruction and mortality and proposed an algorithm for clinical use [8]. To apply this concept to case finding, and in line with the Lancet Commission Report’s call for an expanded definition of COPD [9], we applied an algorithm based on the COPDGene algorithm to stratify smokers into COPD risk phenotypes and found that they were related to smoking intensity and expanded the pool of smokers to be stratified [10]. Like COPDGene, our algorithm used the GOLD definition of airflow obstruction. However, because FEV1/FVC tends to decline with age [11], relying solely on the GOLD diagnostic criteria to assess COPD risk may lead to misclassification of older individuals with a more favorable phenotype as having a more severe phenotype. This issue is particularly relevant for individuals aged 60 years and older, a demographic group in which a significant number of individuals suspected of having COPD undergo screening. To address the age-related changes in spirometry, the Global Lung Initiative (GLI) has introduced the LMS (lambda, mu, and sigma) method, which involves calculating z-scores for FEV1, FVC, and FEV1/FVC [12]. Using this method, Vaz Fragoso and colleagues [13–16] demonstrated that the LMS approach yielded clinically significant results in elderly subjects, supporting its utility in the diagnosis of COPD. Their results also suggested that the GOLD diagnostic threshold could potentially misclassify healthy individuals as having respiratory impairment.

To further explore this issue, we used our clinical algorithm [10] to investigate how the GOLD and GLI thresholds might affect the classification into COPD risk phenotypes of smokers aged 60 years and older. This investigation was particularly relevant given the higher prevalence of COPD in older age groups [17] and the potential risks associated with overdiagnosis, such as unnecessary medication use and increased likelihood of adverse effects.

## Study Methods

### Study Population

The source population included 200 subjects aged ≥ 60 years, selected from 864 smokers who participated in a COPD case-finding intervention at the Pulmonary Institute of Shaare Zedek Medical Center in Jerusalem between May 2014 and June 2017, and provided written informed consent [10]. We excluded hospitalized patients, subjects seeking medical care, and subjects unable to perform spirometry. The Helsinki Ethics Committee of Shaare Zedek Medical Center [IRB #16/14 SZMC] approved the study.

### COPD Risk

Since imaging studies could not be performed in the setting of our study, our algorithm included only 3 of the 4 COPDGene parameters, namely.

I) Smoking exposure: assessed by smoking history, including age at first cigarette, number of cigarettes smoked per day, and years of smoking;
II) Respiratory symptoms: assessed by the following 5-item questionnaire developed by the Canadian Thoracic Society [18]: (1) Do you cough regularly? (2) Do you cough up sputum regularly? (3) Do you get short of breath when doing simple tasks? (4) Do you wheeze with exertion or at night? (5) Do you often get colds that last longer than other people you know? A positive answer to any question is considered clinically significant.
III) Spirometry: According to the COPDGene algorithm [8], pre-bronchodilator spirometry was performed as previously described [10], following the recommendations of the American Thoracic Society and the European Respiratory Society [19]. The main measures of interest were forced vital capacity (FVC), forced expiratory volume in one second (FEV1), and FEV1/FVC ratio.

### Definition of Abnormal Spirometry

Spirometry was considered abnormal in the presence of either airflow obstruction defined by GOLD or GLI and/or PRISm spirometry, as follows:

- **Airflow Obstruction**
  1. GOLD criterion: FEV1/FVC < 0.7. Severity was categorized as follows: a. Mild: FEV1 ≥ 80% predicted; b. Moderate: FEV1 < 80% but ≥ 50% predicted; c. Severe: FEV1 < 50% but ≥ 30% predicted; and d. Very severe: FEV1 < 30% predicted [3].
  2. GLI criterion: FEV1/FVC < LLN5. Z-scores for FVC, FEV1 and FEV1/FVC were calculated using the GLI spirometry reference equations. The LLN for each patient was obtained from the official GLI spirometry website (https://vitalograph.com/normalvalues/gli). The LLN was determined based on a single z-score of—1.64, which established the LLN5. The severity of airflow obstruction was classified as 1. Mild: z-score greater than or equal to—1.64; 2. Moderate: z-score less than—1.64 but greater than or equal to—2.55; and 3. Severe: z-score less than—2.55. A z-score of—2.5 corresponds to the 0.5 percentile distribution and—1.64 corresponds to the LLN [15].
- **Preserved Ratio Impaired Spirometry (PRISm)**
  This was characterized by FEV1 < 80% of predicted and FEV1/FVC ratio > 0.70 [20]. Similarly, this index can be expressed based on GLI by considering an FEV1/FVC above LLN5 and an FEV1 below LLN. We call this PRISm GLI.

### Outcomes

Because symptoms and airflow obstruction are characteristics that require treatment (e.g., smoking cessation) to change the outcome (i.e., risk of COPD; lung morbidity), we termed the groups resulting from their mutually exclusive combination ‟clinical risk phenotypes” [21]. Using the 3 disease characteristics, participants were classified into 4 mutually exclusive GOLD and GLI phenotypes, each including all combinations of the 3 disease characteristics, with exposure (i.e., cigarette smoking) considered positive in all phenotypes [8]. (1) Phenotype A: Exposure only and reference phenotype; no COPD; (2) Phenotype B: Respiratory symptoms and normal spirometry; possible COPD; (3) Phenotype C: No respiratory symptoms and abnormal spirometry; possible COPD; and (4) Phenotype D: Respiratory symptoms and abnormal spirometry; probable COPD.

### Statistics

Statistical analysis was performed using Stata (version 16) software (Stata Corp TX). In a first step, the study population divided according to the GOLD-based and GLI-based phenotypes, respectively, was described in terms of demographics, smoking, medical history, current symptoms, spirometry, and severity of obstruction according to both GOLD and GLI. In a second step, the two phenotypes and the two severity scores were cross-tabulated. Finally, the characteristics of subjects classified as ‟probable COPD” by both GLI and GOLD were compared with those classified as ‟probable COPD” by GOLD but not GLI. Comparisons were based on standard *t* tests or ANOVA when more than 2 groups were compared for continuous variables and Fisher’s exact tests for categorical variables. *P* values < 0.05 were considered significant.

## Results

Table 1 shows the baseline characteristics of the GOLD-defined phenotypes. Phenotype D (probable COPD) was the most common, followed by phenotypes B (possible COPD), A (no COPD), and C (possible COPD). Demographic characteristics were similar across phenotypes, except for BMI, which tended to be lower in phenotypes C and D. Smoking patterns were similar across phenotypes, although phenotype D had a higher, non-significant pack-year consumption. Smokers with phenotype D had significantly more reported cases of COPD than the other phenotypes. As expected, subjects classified as phenotypes B and D had more symptoms than those with the other phenotypes, whereas subjects classified as phenotypes C and D had more spirometry abnormalities. Table 2 shows the corresponding data for the GLI-defined phenotypes. While the overall pattern of results is similar to that described for the GOLD-defined phenotypes, there was a shift in the prevalence of the phenotypes, with an increase in the prevalence of the least affected phenotype, phenotype A, and a decrease in the prevalence of the most affected phenotype, phenotype D.

**Table 1:** Distribution of older smokers according to the COPD risk phenotype defined by a combination of smoking exposure, symptoms and spirometry according to gold spirometric thresholds

Symptoms	NO		YES		NO		YES		
Spirometry	Normal*		Normal*		Abnormal¥		Abnormal¥		
Phenotype	GOLD A		GOLD B		GOLD C		GOLD D		*P* value
N, (%)	29	(14.5)	62	(31.0)	23	(11.5)	86	(43.0)	
Age [years]	64.8	(3.7)	65.0	(4.3)	65.5	(4.5)	66.4	(4.8)	0.21
Male *n* (%)	19	(65.5)	43	(69.4)	16	(69.6)	58	(67.4)	
Female *n* (%)	10	(34.5)	19	(30.6)	7	(30.4)	28	(32.6)	0.98
BMI [kg/m^2^]	27.1	(3.3)	26.9	(4.2)	24.5	(3.6)	25.7	(4.2)	< 0.0428
*Smoking*									
Age first cigarette	19.8	(3.2)	21.1	(8.9)	19.4	(8.1)	19.8	(7.2)	0.69
Cigarettes/day	18.4	(9.8)	20.9	(12.6)	22.0	(12.4)	22.6	(13.4)	0.48
Duration years	44.8	(4.9)	42.6	(10.2)	44.5	(7.8)	45.6	(8.8)	0.23
Pack-years	42.5	(21.0)	44.9	(28.9)	51.3	(30.4)	54.0	(32.9)	0.17
*Medical history*									
Allergies *n* (%)	5	(17.2)	10	(16.1)	1	(4.3)	14	(16.3)	0.53
Asthma *n* (%)	0	(0.0)	0	(0.0)	0	(0.0)	4	(4.7)	0.22
Bronchitis *n* (%)	0	(0.0)	1	(1.6)	0	(0.0)	4	(4.7)	0.54
COPD *n* (%)	0	(0.0)	1	(1.6)	0	(0.0)	9	(10.5)	0.038
*Current symptoms*									
Cough *n* (%)	0	(0.0)	30	(48.4)	0	(0.0)	52	(60.5)	0.18
Phlegm *n* (%)	0	(0.0)	36	(58.1)	0	(0.0)	59	(68.6)	0.23
Dyspnea *n* (%)	0	(0.0)	29	(46.8)	0	(0.0)	56	(65.1)	0.03
Wheeze *n* (%)	0	(0.0)	26	(41.9)	0	(0.0)	47	(54.7)	0.14
Frequent Colds *n* (%)	0	(0.0)	13	(21.0)	0	(0.0)	30	(34.9)	0.070
*Spirometry*									
FEV1 [% predicted]	95.9	(12.1)	94.9	(12.4)	68.9	(10.3)	58.6	(15.8)	< 0.001
FVC [% predicted]	94.6	(14.5)	95.6	(12.8)	73.9	(13.4)	70.5	(16.5)	< 0.001
FEV1/FVC [% observed]	79.2	(6.3)	77.0	(6.2)	72.9	(8.3)	64.1	(11.0)	< 0.001

Values are mean (SD) except when otherwise indicated

*FEV1/FVC ≥ 0.7; PRISm = No ¥ = FEV1/FVC < 0.7 and/or PRISm

The definitions of the GOLD phenotypes are as follows:

GOLD A: no symptoms and normal spirometry by GOLD; no COPD

GOLD B: respiratory symptoms and normal spirometry by GOLD; possible COPD

GOLD C: no respiratory symptoms and abnormal spirometry by GOLD; possible COPD

GOLD D: respiratory symptoms and abnormal spirometry by GOLD; probable COPD

**Table 2:** Distribution of older smokers according to the COPD risk phenotype defined by a combination of smoking exposure, symptoms, and spirometry according to GLI spirometric thresholds

Symptoms	NO		YES		NO		YES		
Spirometry	Normal*		Normal*		Abnormal §		Abnormal §		
Phenotype	GLI A		GLI B		GLI C		GLI D		*P* value
*N* (%)	37	(18.5)	84	(42.0)	15	(07.5)	64	(32.0)	
Age [years]	65.7	(4.4)	65.3	(4.5)	63.5	(2.3)	66.4	(4.8)	0.13
Male *n* (%)	22	(59.5)	55	(65.5)	13	(86.7)	46	(71.9)	
Female *n* (%)	15	(40.5)	29	(34.5)	2	(13.3)	18	(28.1)	0.23
BMI [kg/m^2^]	26.7	(3.4)	26.6	(4.1)	24.1	(3.7)	25.6	(4.4)	0.08
*Smoking*									
Age first cigarette	20.2	(5.3)	20.7	(8.3)	18.3	(6.9)	20.0	(7.7)	0.73
Cigarettes/day	18.9	(9.2)	21.5	(13.0)	22.9	(14.7)	22.4	(13.1)	0.56
Duration years	44.8	(4.9)	43.1	(9.3)	45.3	(9.3)	46.4	(7.0)	0.68
Pack-years	42.8	(19.9)	47.7	(31.2)	55.2	(35.6)	53.4	(31.8)	0.29
*Medical history*									
Allergies *n* (%)	6	(16.2)	11	(13.1)	0	(0.0)	13	(20.3)	0.23
Asthma *n* (%)	0	(0.0)	1	(1.2)	0	(0.0)	3	(4.7)	0.46
Bronchitis *n* (%)	0	(0.0)	2	(2.4)	0	(0.0)	3	(4.7)	0.67
COPD *n* (%)	0	(0.0)	2	(2.4)	0	(0.0)	8	(12.5)	0.019
*Current symptoms*									
Regular cough *n* (%)	0	(0.0)	45	(53.6)	0	(0.0)	37	(57.8)	0.61
Regular phlegm *n* (%)	0	(0.0)	53	(63.1)	0	(0.0)	42	(65.6)	0.86
Dyspnea *n* (%)	0	(0.0)	42	(50.0)	0	(0.0)	43	(67.2)	0.044
Wheeze *n* (%)	0	(0.0)	38	(45.2)	0	(0.0)	35	(54.7)	0.32
Frequent colds *n* (%)	0	(0.0)	17	(20.2)	0	(0.0)	26	(40.6)	0.010
*Spirometry*									
FEV1 [% predicted]	92.8	(13.2)	93.3	(12.9)	56.2	(15.2)	56.2	(15.2)	< 0.001
FVC [% predicted]	91.5	(15.3)	93.9	(13.3)	69.5	(17.3)	69.5	(17.3)	< 0.001
FEV1/FVC [% observed]	79.3	(6.6)	77.0	(6.0)	62.5	(10.5)	62.5	(10.5)	< 0.001

Values are mean (SD) except when otherwise indicated

*GLI Z score FEV1/FVC > −1.64, PRISmGLI—No; § = GLI z-score FEV1/FVC < −1.64 or PRISmGLI

The definitions of the GLI phenotypes are as follows:

GLI B: respiratory symptoms and normal spirometry by GLI; possible COPD

GLI A: no symptoms and normal spirometry by GLI; no COPD

GLI C: no respiratory symptoms and abnormal spirometry by GLI; possible COPD

GLI D: respiratory symptoms and abnormal spirometry by GLI; probable COPD

- Cross-tabulation of phenotypes
  Table 3 shows the cross-tabulation of GOLD- and GLI-defined phenotypes. The two methods gave identical classifications on 170 occasions (yellow cases), namely 29 subjects with phenotype ‟A,”, 62 with phenotype ‟B,” 15 with phenotype ‟C,” and 64 with phenotype ‟D.” There were 30 cases of disagreement: 8 subjects were classified as normal phenotype ‟A” by GLI and as phenotype ‟C” by GOLD (blue case) and 22 subjects were classified as phenotype ‟B” by GLI and as phenotype ‟D” by GOLD (green case).

**Table 3:**
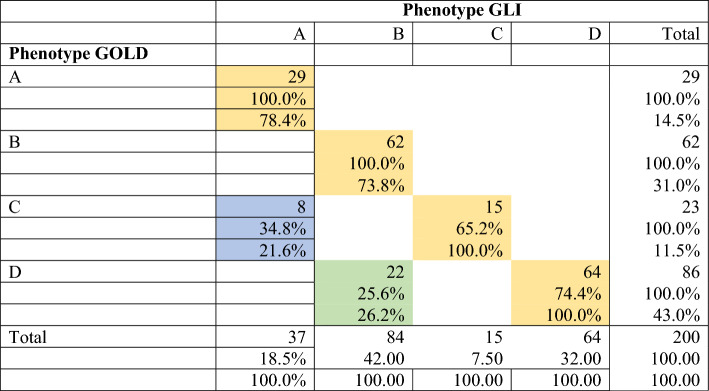
Cross-tabulation of phenotypes obtained according to GLI and GOLD spirometric thresholds

The definitions of the phenotypes are as follows: Phenotype A: no symptoms and normal spirometry (by GLI and GOLD); no COPD; Phenotype B: respiratory symptoms and normal spirometry (by GLI and GOLD); possible COPD; Phenotype C: no respiratory symptoms and abnormal spirometry (by GLI or GOLD); possible COPD; and Phenotype D: respiratory symptoms and abnormal spirometry (by GLI or GOLD); probable COPD
–Comparison of smokers with phenotype D by both GOLD and GLI with those classified by GOLD alone (potential false positives)
  Table 4 shows that among symptomatic patients, those classified as having COPD by GOLD alone, i.e., potential false-positive cases of COPD, did not differ significantly from those classified as ‟probable COPD” by both GOLD and GLI criteria with respect to demographics, smoking, medical history, and symptoms. However, potential false-positive cases of COPD had significantly better average spirometry values than smokers diagnosed with probable COPD by both GOLD and GLI and not a single case of very severe COPD.

**Table 4:** Characteristics of older smokers classified as “Probable COPD” by both GLI and GOLD criteria versus subjects classified as “probable COPD” only by GOLD

	Probable COPD				
	Both GLI and GOLD (*n* = 64)		Only by GOLD (*n* = 22)		*p*
*Demographics*					
Age [years]	66.4	(4.8)	66.2	(4.9)	0.84
Male *n* (%)	46	(71.9)	12	(54.5)	–
Female *n* (%)	18	(28.1)	10	(45.5)	0.19
BMI [kg/m^2^]	25.6	(4.4)	25.9	(3.6)	0.73
*Smoking*					
Age first cigarette	20.0	(7.7)	19.4	(6.0)	0.72
Cigarettes/day	22.4	(13.1)	23.1	(14.3)	0.82
Duration of smoking yr	45.3	(9.3)	46.4	(7.00)	0.59
Pack-years	53.4	(31.8)	55.7	(36.6)	0.79
*Medical History*					
Allergies *n* (%)	13	(20.3)	1	(4.5)	0.10
Asthma *n* (%)	3	(4.7)	1	(4.5)	0.73
Bronchitis *n* (%)	3	(4.7)	1	(4.5)	0.99
COPD *n* (%)	8	(12.5)	1	(4.5)	0.44
*Symptoms*					
Regular cough *n* (%)	37	(57.8)	15	(68.2)	0.46
Regular phlegm *n* (%)	42	(65.6)	17	(77.3)	0.43
Dyspnea *n* (%)	43	(67.2)	13	(59.1)	0.61
Wheeze *n* (%)	35	(54.7)	12	(54.5)	0.99
Frequent colds *n* (%)	26	(40.6)	4	(18.2)	0.07
Nb symptoms	2.9	(1.6)	2.8	(1.1)	0.81
*Spirometry*					
FEV1 [% predicted]	56.2	15.2	84.5	(10.1)	< 0.001
FVC [% predicted]	69.5	(17.3)	92.2	(15.8)	< 0.001
FEV1/FVC [% observed]	62.5	(10.5)	71.5	(5.1)	< 0.001
*Severity Obstruction GOLD*					
No obstruction *n* (%)	0	(0.0)	0	(0.0)	
Mild *n* (%)	2	(4.0)	10	(71.4)	
Moderate *n* (%)	24	(48.0)	4	(28.6)	
Severe *n* (%)	21	(42.0)	0	(0.0)	
Very severe *n* (%)	3	(6.0)	0	(0.0)	< 0.001
PRISm *n* (%)	14	(21.9)	8	(36.4)	0.26
*Severity Obstruction GLI*					
None *n* (%)	0	(0.0)	22	(100.0)	
Mild *n* (%)	23	(57.5)	0	(0.0)	
Severe *n* (%)	17	(42.5)	0	(0.0)	< 0.001
PRISm GLI *n* (%)	24	(37.5)	0	(0.0)	< 0.001

Values are mean (SD) except when otherwise indicated

## Discussion

This study found that the choice between GOLD or GLI diagnostic thresholds in a clinical algorithm affects the classification of older smokers into COPD risk phenotypes. Our results showed discrepancies between GOLD- and GLI-defined phenotypes in 30 out of 200 participants (15%). Importantly, all discrepancies resulted in reclassification of smokers with more severe GOLD-defined phenotypes to less severe GLI-defined phenotypes. Specifically, 8 of 23 (32%) subjects with GOLD-defined phenotype ‟C” (possible COPD) were reclassified to GLI-defined phenotype ‟A” (normal, no COPD), whereas 22 of 86 (25.6%) subjects classified as GOLD-defined phenotype ‟D” (probable COPD) were reclassified to GLI-defined phenotype ‟B” (possible COPD). This pattern is consistent with the hypothesis that the GOLD threshold may misclassify individuals with normal spirometry as having respiratory impairment. If this hypothesis is true, the use of the GOLD-defined threshold could lead to unnecessary and potentially harmful treatment of less affected or unaffected elderly individuals.

The use of the GLI rather than the GOLD spirometric threshold is supported by the fact that the LMS method takes into account age-related changes in lung function. However, strictly speaking, in the absence of a gold standard for obstruction, a simple head-to-head comparison would not allow us to say that one method is superior to the other. Nevertheless, many studies have provided evidence of misdiagnosis of COPD due to the use of the GOLD diagnostic threshold. Our results are consistent with studies in the general and elderly populations. A recent analysis of COPDGene data from more than 10,000 current and former smokers aged 45–80 years with and without COPD and healthy non-smoking controls showed that a severity classification based on the GLI global and 2021 ERS/ATS standards reference equations could discriminate survival, imaging characteristics, and COPD exacerbation rates better than the GOLD classification [22]. In older populations, a study by Hardie and colleagues [23] of healthy individuals aged 70–100 years found that 35% of participants met the GOLD criteria for COPD, despite never having smoked or experienced respiratory symptoms or a previous diagnosis of lung disease. In 4102 individuals aged 60 years and older, Medbo and Melbye [24] found that the FEV1/FVC ratio decreased significantly after the age of 60 years, even in healthy non-smokers. They concluded that the use of the 70% threshold for all ages is an oversimplification and called for an adjustment of the GOLD criteria for the diagnosis of COPD. Based on their data, they suggested that FEV1/FVC ratios as low as 65% should be considered normal in subjects aged 70 years or older. In a large sample of individuals aged 40–80 years [*n* = 3502], Vaz Fragoso and colleagues [13] found that an LMS-LLN threshold for FEV1/FVC at the fifth percentile of the z-score distribution (i.e., LMS-LLN5) was associated with an increased risk of death and the likelihood of having respiratory symptoms, supporting its use as a basis for identifying COPD in middle and older age. Later, the same team found that GLI-defined normal spirometry, even when classified as obstructive by GOLD, included adjusted means in the normal range for several respiratory-related phenotypes, including dyspnea severity, health-related quality of life, 6-min walk test, bronchodilator reversibility, percentage of lung with emphysema and gas trapping as measured by computed tomography, and small airway dimensions [15]. Finally, the same team [16] showed that there was a graded association between GLI-defined spirometric impairment, including mild, moderate, and severe COPD and restrictive pattern and the above respiratory phenotypes. Taken together, these studies suggest that the diagnostic threshold published by GOLD often misidentifies COPD and overestimates its prevalence with advancing age.

Our results are in contrast to the study by Bhatt and colleagues [25], which supports the use of the GOLD fixed ratio criterion to identify subjects at risk for COPD. However, this study differs from ours in many ways that preclude a direct comparison. First, approximately half of the population in Bhatt’s study (~ 12,000 of 24,000) was younger than 60 years and second, Bhatt’s study defined COPD by spirometry—either GOLD or LLN threshold—which is the opposite of the expanded definition advocated by COPDGene and used in our study. More importantly, whereas our study classified smokers into risk phenotypes ranging from *no disease* to *possible disease*, Bhatt and colleagues sought to determine the discriminatory accuracy of different FEV1:FVC thresholds for predicting COPD-related *hospitalization and mortality*, i.e., patients with advanced disease. In addition, our study used the COPDGene concept of abnormal spirometry, which includes PRISm spirometry, whereas Bhatt’s study defined abnormal spirometry based only on the FEV1/FVC ratio. Finally, unfortunately, in their search for the optimal FEV1:FVC threshold, Bhatt and colleagues investigated a single threshold for the GLI z-score.

The results of this study are clinically relevant. When we compared subjects with probable COPD defined by both GOLD and GLI (*n* = 64) with subjects with probable COPD defined by GOLD alone (*n* = 22) (i.e., potential false-positive cases), we found that, consistent with misclassification, the potential false positives had significantly better mean baseline spirometric values and no cases of airflow obstruction or PRISm spirometry (Table 4). In addition, the use of an expanded definition of COPD increased the population of smokers to be screened. Because they do not have GOLD-defined obstruction, smokers classified as phenotype B (possible COPD) would be considered ‟without COPD” according to current guidelines [26], although they have symptoms that warrant clinical evaluation. According to our approach, these smokers should be offered more sensitive tests to detect airflow obstruction rather than being told they have ‟normal” spirometry [27]. Second, because they are asymptomatic, phenotype ‟C” (‟possible COPD”) smokers would be considered ineligible for screening according to current guidelines [26], so their abnormal spirometry would go undetected. In our opinion, the correct work-up for these smokers should consist of a more thorough examination of symptoms using more detailed questionnaires and, if necessary, an assessment of exertional dyspnea [28]. In total, this potentially missed population amounts to 85 subjects classified using the GOLD diagnostic threshold (i.e., 60 as GOLD B + 23 as GOLD C) or 99 subjects classified using the GLI diagnostic threshold (84 as GLI B + 15 as GLI C) (Table 1). As a quick aside, telling these smokers that they ‟do not have COPD” could be perceived as a license to smoke and delay smoking cessation for years.

A limitation of this study is the lack of imaging studies that would have allowed classification of smokers into the ‟definite COPD” phenotype [7, 8]. However, a definitive diagnosis of COPD in subjects enrolled in a case-finding intervention is usually made on an individual basis after the case-finding intervention. In addition, obtaining lung imaging would have made this study too expensive and impractical and would have discouraged participation. According to the COPDGene algorithm, we performed only pre-BD spirometry [8]. Reasons for not performing BD testing in case finding include diurnal variation in FEV1, lack of reproducibility, and arbitrary definition of a significant test [29]. Furthermore, excluding all subjects with a positive BD test could lead to under-diagnosis of COPD, as some patients may have true reversibility independent of asthma [30].

## Conclusion

In conclusion, this study suggests that the use of GOLD-defined diagnostic thresholds in a clinical algorithm classified older smokers into more severe COPD risk phenotypes than the use of GLI-defined thresholds, supporting the idea that GOLD may misclassify subjects with a normal or less affected phenotype as having respiratory impairment [13–16]. This has clinical implications, as COPD is a heterogeneous disease and spirometry, along with other parameters, will remain essential for diagnosis [7–9]. Misclassification of elderly smokers into severe COPD phenotypes could lead to inappropriate and harmful treatment. While the economic cost of such misclassification is difficult to estimate, the psychological impact on misdiagnosed individuals could be significant. In addition, this study supports previous research suggesting that classifying smokers into risk phenotypes, rather than based solely on FEV1/FVC < 0.7, may increase the population requiring screening and support preventive measures such as smoking cessation [7, 10]. Further longitudinal studies are needed to validate these findings.

## Data Availability

All participant-level data relevant to the study are included in the article. Study data are available from the corresponding author upon reasonable request, after removal of all personal identifiers, and after approval by the Shaare Zedek Medical Center Ethics Committee.

## Abbreviations

COPD: Chronic obstructive pulmonary disease
GOLD: Global initiative for obstructive lung disease
GLI: Global lung initiative
FEV1: Forced expiratory volume in one second
FVC: Forced vital capacity
LMS: Lambda, Mu, Sigma
PRISm: Preserved ratio impaired spirometry
LLN: Lower limit of normal
